# Intralesional Talimogene Laherparepvec Immunotherapy for Melanoma: A Case Study

**DOI:** 10.7759/cureus.69605

**Published:** 2024-09-17

**Authors:** Merhawit Ghebrehiwet, Janelle Pavlis, Carlos Gomez-Meade

**Affiliations:** 1 College of Medicine, Oklahoma State University Center for Health Sciences, Tulsa, USA; 2 Dermatology, Oklahoma Cancer Specialists and Research Institute, Tulsa, USA

**Keywords:** imlygic, melanoma, oncolytic virotherapy, talimogene laherparepvec, t-vec

## Abstract

Melanoma, characterized by its aggressive nature and tendency for metastasis, presents a significant challenge in clinical management. While surgical excision remains the gold standard for localized disease, therapeutic advancements for advanced stages are crucial. Oncolytic virotherapy, exemplified by Talimogene laherparepvec (T-VEC), offers a potential approach. Here, we present a 79-year-old male with advanced melanoma on the left distal thumb who opted for T-VEC therapy over surgical excision. Over 18 sessions, T-VEC demonstrated efficacy, resulting in lesion regression and no evidence of recurrence upon long-term monitoring. This case emphasizes the potential of T-VEC in advanced melanoma management, leveraging its dual mechanism targeting primary and metastatic lesions while harnessing immune response. Safety considerations and further investigations into potential interactions with other immunotherapies are warranted to optimize treatment strategies and ensure patient well-being. As oncolytic virotherapy continues to evolve, T-VEC stands as a potential option for the treatment of advanced melanoma.

## Introduction

Melanoma, a malignant tumor originating from melanocytes, poses a significant public health concern globally due to its increasing incidence rates and potential for metastasis and death. According to recent epidemiological data, melanoma accounts for approximately 1%-2% of all skin cancers but is responsible for the majority of skin cancer-related deaths, emphasizing its clinical significance [1].While surgical excision remains the gold standard for curative treatment of localized disease, therapeutic strategies for advanced melanoma have evolved over the past few decades. The inclusion of immunotherapy and targeted therapy has revolutionized the treatment of melanoma, offering improved outcomes and prolonged survival for patients with unresectable or metastatic disease [2].

Among the novel therapeutic methods, oncolytic virotherapy has emerged as an approach for melanoma treatment. Talimogene laherparepvec (T-VEC; IMLYGIC®), a genetically modified herpes simplex virus type 1 (HSV-1), represents a substantial advancement in oncology. T-VEC was approved by the US Food and Drug Administration (FDA) in 2015 and is the first oncolytic viral therapy approved for the treatment of melanoma lesions in the skin and lymph nodes [3].The mechanism of action involves direct tumor lysis and the induction of systemic immunity. T-VEC is engineered to specifically reproduce within and destroy tumor cells, all the while stimulating both local and widespread anti-tumor immune responses [3]. Clinical trials have demonstrated its efficacy in achieving durable responses and improving overall survival in patients with advanced melanoma, especially those with injectable cutaneous and subcutaneous lesions [4]. In this case study, we present a patient with advanced melanoma who experienced a favorable response to T-VEC therapy.

## Case presentation

A 79-year-old male with recently diagnosed unspecified malignant melanoma affecting the left distal thumb was referred to the Mohs surgery clinic in May of 2021 to discuss treatment options. The referring dermatologist suspected tumor staging to be 3, but the patient declined a lymph node biopsy to confirm. The patient endorsed a past medical history of hypercholesterolemia and current medications included aspirin, atorvastatin, furosemide, and potassium chloride. He had no previous history of diagnosed skin cancer.

**Figure 1:**
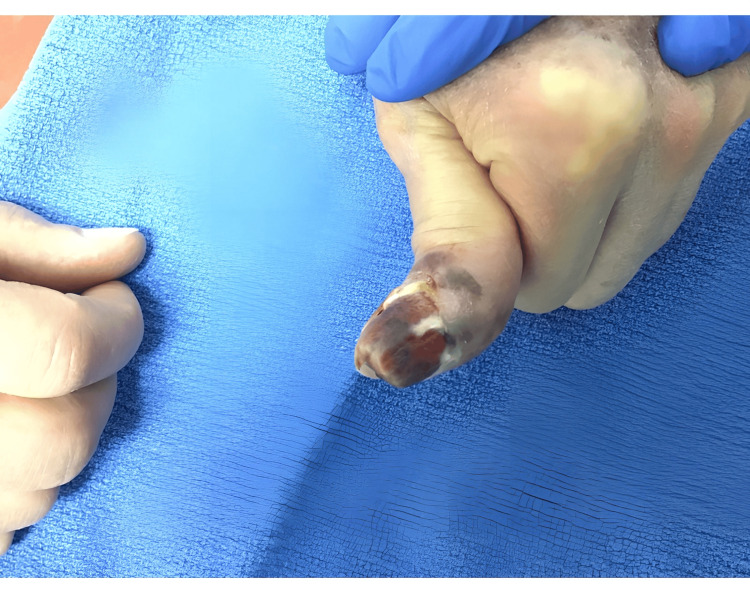
Left distal thumb on initial presentation

The patient requested an alternative treatment from surgical excision due to concerns about losing his thumb from surgical digital amputation. The irregular, pigmented, and ulcerated lesion measured 2.5 cm X 3.0 cm (Figure 1). An initial PET scan during which 14.60 mCi of fludeoxyglucose (FDG) was injected intravenously showed nonspecific uptake in a subcentimeter left axillary lymph node with standardized uptake values (SUVs) peaking at 2.6. The option of neoadjuvant T-VEC therapy was proposed to the patient, and he agreed to try the therapy. A total of one milliliter of T-VEC 10^6^ plaque-forming units (PFU) was injected intralesionally in the left thumb and he tolerated the first injection well without any complications or side effects. The patient was instructed to remain in the clinic for observation for 15 minutes after the injections. The injected area was covered with sterile bandaging, non-adhering and absorbent dressing, and then covered with transparent medical dressing. After two weeks, an ultrasound demonstrated the left axillary nodes remained unchanged in size and no pathologic adenopathy was appreciated.

**Figure 2:**
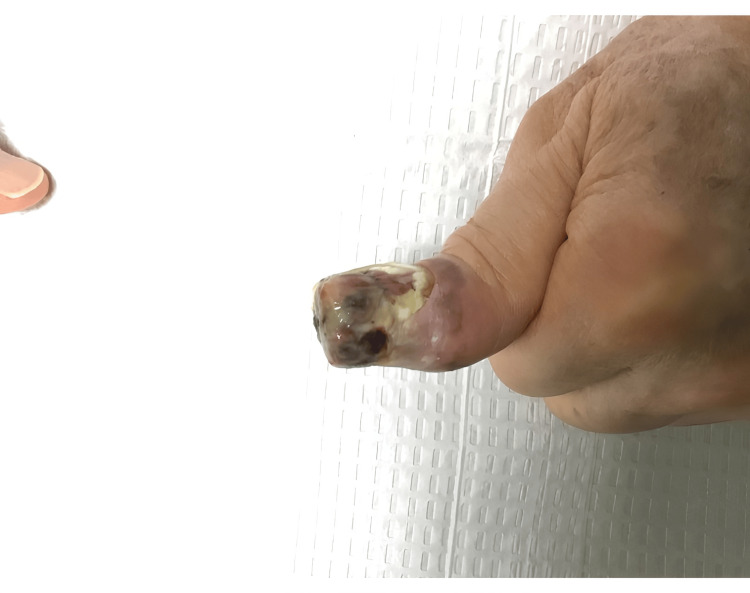
Reactive changes noted following the second intralesional injection

**Figure 3:**
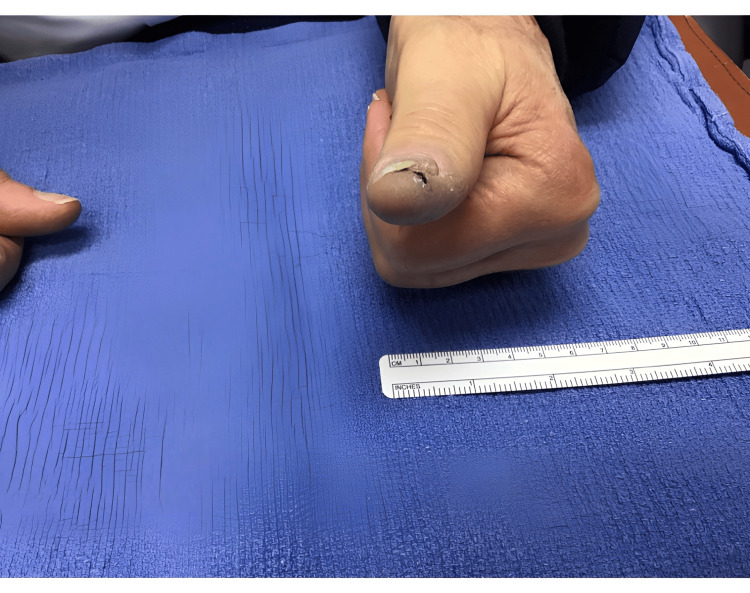
Day of final intralesional injection. A well-healed scar is appreciated

**Figure 4:**
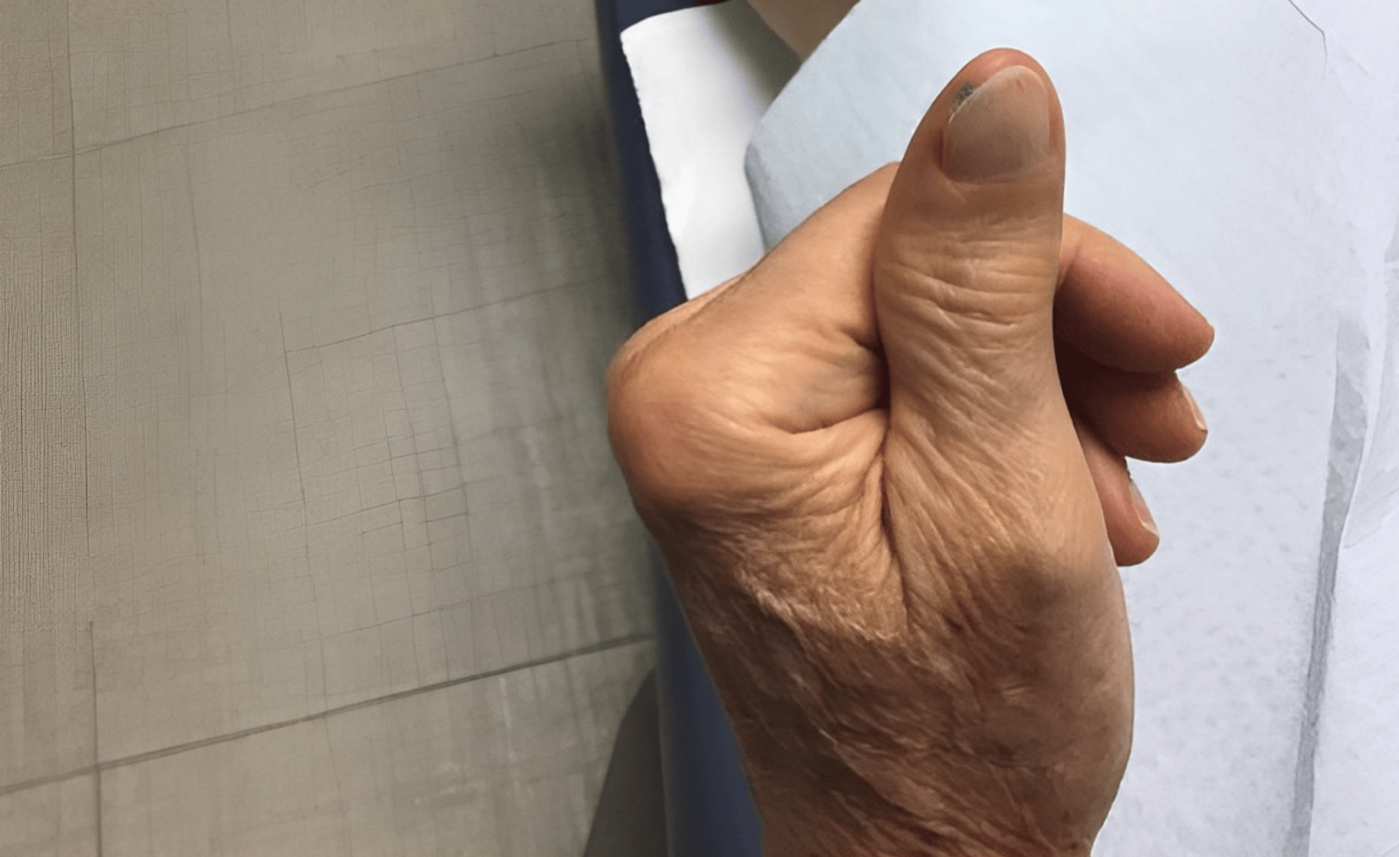
Left distal thumb sixteen months after completing intralesional treatment with T-VEC T-VEC: talimogene laherparepvec

Follow-up assessments, including ultrasounds, demonstrated unchanged sizes of the left axillary nodes, alongside a clinical reduction in black pigment on the thumb several weeks post-initiation of T-VEC therapy (Figure 2). The injections occurred on a biweekly basis for a total of 18 sessions. The pigment continued to gradually decrease with each injection session, and the patient tolerated each injection well. A punch biopsy 10 months after the onset of therapy revealed nodular aggregates of melanophages, inflamed cicatricial fibrosis, and telangiectasia. There was a lack of atypical melanocytic proliferation, suggestive of lesion regression. Subsequent PET imaging indicated no evidence of recurrent disease. Treatment was promptly discontinued based on favorable clinical and histological responses. During the final injection session, the left thumb presented with a well-healed scar (Figure 3). Long-term monitoring plans included skin exams every three months and annual PET scans for the following three years (Figure 4).

## Discussion

The efficacy of T-VEC therapy observed in our case report demonstrates the potential of oncolytic virotherapy as a therapeutic option for advanced melanoma. The dual mechanism of T-VEC targets the primary tumor and elicits an immune response capable of recognizing and attacking metastatic lesions. This approach aligns with the evolving understanding of melanoma as an immunogenic tumor, emphasizing the importance of employing the host immune system as a primary defense [5]. Additionally, the favorable safety profile of T-VEC, characterized by adverse events such as flu-like symptoms and injection-site reactions, suggests an appeal as a therapeutic option for suitable candidates [4]. Beekman et al. presented several cases of HSV-1 dissemination during T-VEC therapy; however, the mentioned patients were previously on immune checkpoint inhibitor therapy [6]. This prompts a critical consideration for future investigations to understand the potential interplays between immunotherapeutic treatments and viral dissemination risks. Further inquiries could benefit patients by optimizing advanced melanoma management strategies while ensuring safety. 

In addition to the current findings, recent studies have suggested the synergistic effects of combining T-VEC with other immunotherapies, such as immune checkpoint inhibitors. This combination has shown promise in enhancing antitumor immune responses and improving clinical outcomes in patients with advanced melanoma. For instance, a study by Chesney et al. reported that the combination of T-VEC and pembrolizumab resulted in higher response rates compared to either treatment alone, highlighting the potential for integrated therapeutic approaches [7]. Moreover, ongoing clinical trials are investigating the efficacy of T-VEC in combination with various systemic therapies, which could lead to more comprehensive treatment protocols for melanoma patients [8]. The integration of T-VEC into multimodal treatment regimens may provide a more comprehensive and sustained antitumor effect, potentially overcoming the limitations of therapies.

Furthermore, the evolving landscape of oncolytic virotherapy emphasizes the need for personalized treatment approaches tailored to individual patient profiles. Factors such as tumor genetics, immune status, and previous treatments can significantly influence the response to T-VEC therapy. Tumor genetics, particularly mutations in the v-raf murine sarcoma viral oncogene homolog B1 (BRAF), Neuroblastoma Ras viral oncogene homolog (NRAS), and receptor tyrosine kinase proto-oncogene (c-KIT) genes, can impact how effectively T-VEC targets and destroys cancer cells. For instance, the presence of BRAF mutations has been associated with variable responses to T-VEC, suggesting that genetic profiling of tumors can be crucial in predicting patient outcomes [9]. Future research should focus on identifying biomarkers that can predict response to oncolytic virotherapy, thereby enabling more precise patient selection and optimizing therapeutic outcomes. Additionally, understanding the long-term effects and potential resistance mechanisms associated with T-VEC will be crucial in refining treatment protocols and enhancing the durability of responses. As ongoing research continues to clarify the workings of oncolytic virotherapy, T-VEC holds potential as a functional addition to the therapeutic options for advanced melanoma disease.

## Conclusions

In conclusion, this case report highlights the efficacy of T-VEC in the treatment of advanced melanoma. T-VEC's dual mechanism of action, involving direct tumor lysis and immune system activation, aligns with the evolving understanding of melanoma as an immunogenic tumor. The favorable safety profile observed in this case study, despite some documented adverse events, suggests that T-VEC is an appealing therapeutic option for eligible patients. However, the potential risks of viral dissemination, especially in patients with prior immune checkpoint inhibitor therapy, necessitate further investigation for optimized treatment strategies and patient safety. As ongoing research advances our understanding, T-VEC stands as a potential addition to the range of options against advanced melanoma, offering hope for improved outcomes and prolonged survival.
